# Evaluation of alarm notification of artificial intelligence in automated analyzer detection of parasites

**DOI:** 10.1097/MD.0000000000039788

**Published:** 2024-09-27

**Authors:** Zila Wang, Lin Liao, Ximei Huang, Jinguang Tang, Faquan Lin

**Affiliations:** a Key Laboratory of Clinical Laboratory Medicine of Guangxi Department of Education, Department of Clinical Laboratory, The First Affiliated Hospital of Guangxi Medical University, Nanning, Guangxi, China.

**Keywords:** artificial intelligence, automated feces analyzer, deep learning machinery, diagnosis, intestinal parasites

## Abstract

To evaluate the alarm notification of artificial intelligence in detecting parasites on the KU-F40 Fully Automatic Feces Analyzer and provide a reference for clinical diagnosis in parasite diseases. A total of 1030 fecal specimens from patients in our hospital from May to June 2023 were collected, and parasite detection studies were conducted using the KU-F40 automated feces analyzer (normal mode method, floating-sedimentation mode method), acid–ether sedimentation method, and direct smear microscopy method, respectively. The positive detection rate of parasites in the 1030 fecal specimens was 22.9% (236 cases), of which the KU-F40 normal mode method had a detection rate of 16.3% (168 cases), the acid–ether sedimentation method had a detection rate of 19.0% (196 cases), and the direct smear microscopy method had a detection rate of 13.1% (135 cases). The detection rates of the first 2 methods were higher than those of the direct smear microscopy method, and the difference was statistically significant (*P* < .05). The detection rate of the KU-F40 floating-sedimentation mode method was 11.9% (123 cases), which was lower than that of the direct smear microscopy, and the difference was not statistically significant (*P* > .05). The sensitivity of the KU-F40 normal mode method, acid–ether sedimentation method, direct smear microscopy method, and the KU-F40 floating-sedimentation mode method were 71.2%, 83.1%, 57.2%, and 52.1%, respectively, and the specificity was 94.7%, 100%, 100%, and 97.7%, respectively. The coincidence rates of the KU-F40 normal mode method was 90.78%, with Kappa values of 0.633. The positive detection rate of parasites using the KU-F40 normal mode method is higher than that using the direct smear microscopy method. It has high sensitivity and specificity and has advantages such as high automation and fast detection speed. It can replace the microscopy method for routine screening and has higher clinical application value in the diagnosis of intestinal parasitic diseases.

## 1. Introduction

Parasitic diseases can affect multiple systems of the human body and cause various fatal diseases, such as malaria, giardiasis, and amebiasis.^[1]^ Parasite morphological examination of biological samples using conventional light microscopy is the gold standard for parasitic disease diagnosis.^[2]^ However, various factors could lead to inaccurate results such as the limitations of workers’ experience and knowledge, the host immunity, and the complexity of the life cycle changes of parasites. These factors make the direct microscopy method not ideal regarding its sensitivity and specificity in some parasitic diseases,^[3]^ leading to incorrect diagnosis and missed diagnoses. Thanks to the continuous improvement of artificial intelligence (AI), the diagnostic methods of parasitic disease have developed rapidly, and detection methods have shifted to aim with higher sensitivity and specificity, resulting in the combinations of many new joint diagnostic methods.^[4]^ In this study, the KU-F40 uses AI deep learning algorithms to automatically identify parasites, which can improve the parasite detection rate and detection efficiency. In this study, we compared the results from the direct smear microscopy method, the acid–ether sedimentation method, and the KU-F40 (normal mode, floating-sedimentation mode) method regarding each method’s detection rate, sensitivity, and specificity by detecting parasites on the same specimen. Our aim was to evaluate the AI alarm notification of the KU-F40 in the detection of parasites.

## 2. Materials and methods

### 2.1. Patients

A total of 1030 fecal specimens from patients of our hospital were collected from May 2023 to June 2023. The same specimen was numbered using a blind method, and different methods were used for parasite detection, including the acid–ether sedimentation method, direct smear microscopy method, and the KU-F40 (normal mode, floating-sedimentation mode) method.

### 2.2. Instruments and reagents

**Figure 1.**
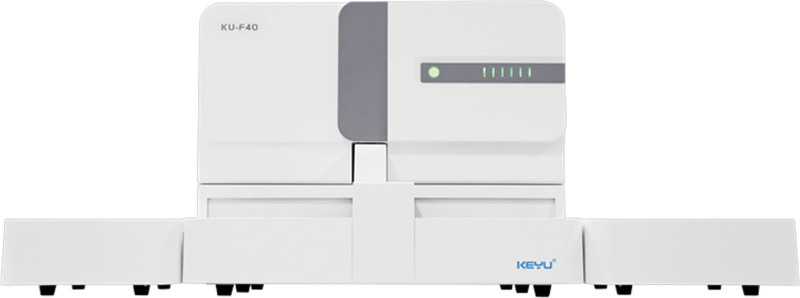
KU-F40 Fully Automatic Feces Analyzer.

The analyzer (Fig. 1), supporting reagents, and consumables were provided by the manufacturer Zhuhai Keyu Bioengineering Co., Ltd. The optical microscope was used; the direct smear microscopy method was applied by using 0.9% normal saline, 50% hydrochloric acid (press 1:1 concentrated hydrochloric acid and distilled water), and diethyl ether.

### 2.3. KU-F40 Fully Automatic Feces Analyzer operation steps

1. Starting up:
  1. Press the switch, then press the start button.
  2. Double click on the desktop KU-F40 icon.
  3. Enter username and password to log in to the software.
  4. Wait for the start button to light up.
2. Colloidal gold reagent card settings:
  1. Click < Management>→<Inspection Items>→<Inspection Item Settings>→<Colloidal Gold>→<Card Box Settings > to add/clear colloidal gold reagent cards.
  2. After setting up the colloidal gold project in the < Card Box Settings>, open the compartment door and place the corresponding reagent card in card boxes 1 to 6. Close the door to activate the colloidal gold project.
3. Specimen testing:
  1. Place the collection cup of the specimen to be tested into a dedicated conventional specimen rack, and then place it into the sample tray.
  2. Click < Management>→<Inspection Items>→<Inspection Item Settings>→ Check < Tangible Ingredients > and < Colloidal Gold>.
  3. In the “Sample Detection” interface, click < Start > to perform the detection.
4. The samples were reexamined:
  1. Place the retest sample collection cup into the dedicated retest sample rack and place it on the sampling tray.
  2. Repeat steps 2 and 3 in step (3).
5. Samples information editing, review and printing.
  1. Select a sample in the “Sample Testing” interface, enter patient information, and save the information,
  2. Multi image review,<Review Confirmation > button
  3. Large image review,<Review Confirmation > button
  4. Select and print sample results
6. Turn off the machine:
  1. Click “Exit”.
  2. Take out the unused colloidal gold reagent card from the card box and place it in a dry and sealed bag.
  3. The operating system Shutting down.

### 2.4. Research method

#### 2.4.1. KU-F40 normal mode method

Prepared fecal specimens around soybean-sized and added them to the sample collection cups; then the instrument automatically took 520 images with the low-magnification lens and 20 images with the high-magnification lens for specimen detection; activated the dilution ratio function which is the system automatically adds diluent based on specimen volume.

#### 2.4.2. KU-F40 floating-sedimentation mode method

Prepared fecal specimens around soybean-sized and added them to the sample collection cups; combined with 2 detection modes, the camera captured 270 images under the low-magnification lens and 20 images under the high-magnification lens each time. Prepared 6 mL of high-concentration saline in the system, and no further diluent was required for the system.

#### 2.4.3. Acid–ether sedimentation method

Poured about 3 mL of 50% hydrochloric acid into the test tube; collected peanut-sized feces by using an applicator stick and placed it in the test tube; stirred evenly and eliminated the feces residue by using an applicator stick. Then about 2 mL of ether was added into the test tube and plugged in a rubber stopper at the top of the test tube. Shaked vigorously for about 20 seconds then slowly removed the rubber stopper. The test tube was centrifuged at 2500 rpm for 3 minutes. Then the content in the test tube was separated into 4 layers: ether, fatty feces, hydrochloric acid, and sediment layers. Removed the upper 3 layers and added about 5 mL of distilled water to wash the sediment layer. After washing, centrifuged the test tube and poured off the supernatant. Only the sediment was used for microscopic examination. Observed the supernatant under microscopy by first using a low-power lens to look for intestinal parasite eggs and protozoal cysts; and then switching to a high-power lens to distinguish and confirm the parasites.

#### 2.4.4. Direct smear microscopy method

Added 1 to 2 drops of normal saline in the center of the microscope slide; obtained the soybean-size feces with an applicator stick and emulsified in the saline. The direct smear should be thin enough so that a printed page can be read through it. Observed under the low-power lens to examine suspicious eggs, then switched to a high-power lens for better observation and confirmation of the parasites.

### 2.5. Statistical methods

SPSS 23 software was used for data analysis and processing. Enumeration data were expressed as the number of cases or rate (%), *P* < .05 was used to indicate statistically significant differences. The sensitivity, specificity, and kappa agreement are presented as a proportion with 95% confidence interval (CI), and a comparison between the 2 groups was performed using the McNemar test.

## 3. Results

### 3.1. Comparison of parasite detection rates among 4 detection methods

**Figure 2.**
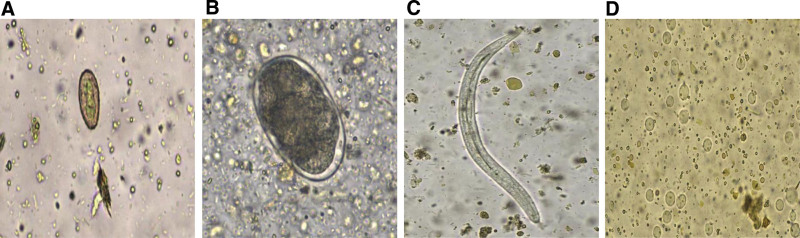
Images of various parasites. (a) *Clonorchis sinensis*, (b) *hookworm*, (c) *Fecal matter Strongyloidiasis*, and (d) *Blastocystis hominis*.

After conducting different parasite examination methods on the 1030 patients, a total of 236 positive cases were detected, including 231 cases of *Clonorchis sinensis*, including 2 cases of mixed infection (*Clostridium sinensis* combined with *hookworm*, *C sinensis* combined with *fecal matter Strongyloidiasis*); 4 cases of *hookworm* and 1 case of *Blastocystis hominis* (Fig. 2). As shown in Table 1, the number of positive cases detected by the direct smear microscopy method was 135 cases, the acid–ether sedimentation method was 196 cases, and the 2 detection methods of the KU-F40 (normal mode and floating-sedimentation mode) method were 168 cases, 123 cases, respectively. The detection rate of direct smear microscopy was 13.1%, which was lower than the acid–ether sedimentation method (19.0%) and the KU-F40 normal mode method was 16.3% (*P* < .05), while the detection rate of the KU-F40 floating-sedimentation method (11.9%) was lower than that of the direct smear microscopy method (*P* > .05). There was statistically significant difference between the acid–ether sedimentation method and the KU-F40 normal mode method (*P* < .05). At the same time, comparing the sensitivity, specificity, and Youden index of the 4 methods, the diagnostic value of the acid–ether sedimentation method and the KU-F40 normal mode method was better than that of the direct smear microscopy method and the KU-F40 floating-sedimentation mode method.

**Table 1:** Comparison of positive detection rate, sensitivity and specificity of 4 different methods (n = 1030).

Detection method	Number of positive cases	Positive detection rate (%)	Sensitivity (%)	Specificity (%)	Youden Index	Positive predictive value (%)	Negative predictive value (%)
Direct smear microscopy method	135	13.1	57.2	100	0.57	100	88.72
Acid–ether sedimentation method	196	19.0	83.1	100	0.83	100	95.20
KU-F40 normal mode method	168	16.3	71.2	94.7	0.71	100	92.11
KU-F40 flotation-sedimentation mode method	123	11.9	52.1	97.7	0.52	100	87.54

### 3.2. Comparison of the results of 4 different methods for detecting parasites

Taking the direct smear microscopy method as the standard method (Table 2), the total coincidence rates of the acid–ether sedimentation method, KU-F40 normal mode method, and KU-F40 floating-sedimentation mode method were 90.19%, 90.78%, and 91.07% respectively. A Kappa test was performed on the consistency of the results. The Kappa values of the acid–ether sedimentation method and the KU-F40 normal mode method were 0.639 (95% CI 0.574–0.704) and 0.633 (95% CI 0.566–0.70), respectively, both of which were highly consistent. The Kappa value of the KU-F40 floating-sedimentation method was 0.592 (95% CI 0.518–0.666), which was good. There was moderate consistency, and the differences were statistically significant (*P* < .05).

**Table 2:** Analysis of the results of 4 different methods for detecting parasitic eggs (n).

Method		Direct smear microscopy		Total	Coincidence rate	Kappa value
		Positive	Negative			
Acid–ether sedimentation method	Positive	115	81	196	90.19	0.639
	Negative	20	814	834		
	Total	135	895	1030		
KU-F40 normal mode method	Positive	104	64	168	90.78	0.633
	Negative	31	831	862		
	Total	135	895	1030		
KU-F40 Flotation-sedimentation mode method	Positive	83	40	123	91.07	0.592
	Negative	52	855	907		
	Total	135	895	1030		

### 3.3. Assessment of the AI alarm notification to detect parasites in the KU-F40

Among the 1030 specimens, a total of 175 positive cases were detected by the KU-F40 normal mode method (Table 3). After being reviewed by experts in testing parasites, the actual number of true positive cases was 168, and the instrument missed 7 cases. The sensitivity of the AI alarm notification was 96% (95% CI 0.916–0.982) and the specificity was 90.4% (95% CI 0.882–0.923).

**Table 3:** KU-F40 Sensitivity and specificity of AI alarm notification.

Method	Cases of true positive	Detection of positive	Miss count	Sensitivity (%)	Specificity (%)
KU-F40 normal mode method	168	175	7	96.0	90.4
KU-F40 floating-sedimentation mode method	123	136	13	90.4	97.2

### 3.4. Assessment of the ability of AI alarm technology to identify liver fluke eggs

Four detection methods were used to count liver fluke eggs. After being reviewed by experts in testing parasites, the true number of liver fluke eggs was obtained. The ability of the KU-F40 to identify liver fluke eggs was then evaluated upon the comparison of the obtained result. AI identified liver fluke eggs. The precision rate of detection of fluke eggs by AI was 93.40%, and the recall rate was 89.40% (Table 4).

**Table 4:** Evaluation of the ability of KU-F40 AI alarm technology to identify liver fluke eggs.

AI recognition	Actual number of liver fluke eggs		Total
	Positive	Negative	
Positive	1267	89	1356
Negative	151	–	–
Total	1418	–	–
Accurate rate		93.40%	
Recall rate		89.40%	

*Note*: The actual number of liver fluke eggs is the number of liver fluke eggs confirmed by parasite testing experts.

## 4. Discussion

In clinical examination, a feces test is a diagnostic tool that is frequently seen in regular test items due to its easy-to-access and noninvasive. It can be used for the etiological examination of various diseases, and it has great significance for the diagnosis of digestive parasitic diseases. Clinically, the key to rapid diagnosis and treatment lies in the accurate detection of parasites. Clinically, using light microscopy is the gold standard for parasite examination and diagnosing parasitic diseases, but it has many weaknesses such as the limitations of workers’ experience and knowledge, the types of parasites, and the complexity of the life cycle changes of parasites, these factors could directly affect the examination result by incorrect diagnosis and misdiagnosis. Hence, applying the light microscopy method to parasite examination still has problems with low sensitivity and high false negative rates.^[2,5]^ To evaluate the AI alarm notification of 2 methods on KU-F40 (normal mode, floating-sedimentation mode), we performed 4 different detection methods on parasite detection.

In this study, we found that all 4 detection methods have missed detections. Compared with the other 3 methods, the acid–ether sedimentation method has the highest positive detection rate, which may be related to the fact that this method requires 2 centrifugations and has a good degree of enrichment. However, the acid–ether sedimentation method has the problems of tedious and time-consuming operation steps, and ether is harmful to human health, so it is not suitable for pathogen screening of large-scale clinical specimens. This study shows that the detection rates of the KU-F40 normal mode method and the acid–ether sedimentation method are relatively close, and their detection efficiency is better than the direct smear microscopy method. The automated feces analyzer has a better detection rate and efficiency of parasites than the light microscopy method^[6,7]^ and it has the advantages of reducing human errors and high biological safety. A survey and analysis of the status of human parasites in China from 2015 showed that the Guangxi region had the highest *C sinensis* infection rate, reaching 16.45%.^[8]^ The same result was obtained by Lu’s team^[9]^ according to their 4-year study. Although the overall infection rate showed a downward trend during the 4 years, the infection rate was still high, and it is still a big concern in the Guangxi region. In this study, the alarm notification of the KU-F40 normal mode method shows a good detection rate and low recall rate. In contrast, the KU-F40 floating-sedimentation mode method shows a low detection rate of liver fluke, this might be due to the high density of liver fluke eggs.

Target detection is a new AI method for identifying and labeling parasitic pathogen morphology. With the development of deep learning in the field of target detection, microscopic images rely on the convolutional neural network.^[10]^ Through weight sharing, the complexity of the model can be reduced, and it improves the generalization performance of the network. Currently, the main detection methods are mainly divided into 2 categories: single-stage model and two-stage model. Among them, the two-stage models mainly include Faster RCNN, and the single-stage models mainly include the YOLO series, SSD series, etc. At the same time, with the continuous development of the YOLO series models, the accuracy of the single-stage model has been greatly improved while maintaining the advantage of speed.

In this study, the KU-F40 adopts the improved single-stage YOLOv5 model. YOLOv5 model is a deep-learning target detection algorithm, it has a great impact on parasitic detection with high accuracy and efficiency. Besides that, accuracy and stability are the keys to an automatic detection analyzer. The method of model identification of parasites is constructed as follows: pretreatment of feces specimens; the construction of a parasite database, in other words, microphotography of parasites in specimens, using different processing techniques to extract the morphological characteristics of image parasite eggs, such as VC^++^ technology,^[11]^ digital image processing technology,^[12]^ phase coherent technology,^[13]^ etc. Classifiers are constructed based on various algorithms to establish parasite identification methods. The cloud-based deep learning algorithm of the VETSCAN IMAGYST system, studied by Nagamori et al,^[14]^ shows similar results to the detection results of parasite experts, and this system can locate, classify, and identify a variety of parasites, and complete the detection within 15 minutes. Naing et al^[15]^ have high sensitivity, specificity, and accuracy in the automatic identification of fecal intestinal parasites, and the detection speed is faster and takes up less memory. Compared with traditional machine learning methods, deep learning methods have good results in parasite detection and classification, but due to the scarcity of parasite image databases, methods and models still have limitations.

The KU-F40 Fully Automatic Feces Analyzer that used in this study can detect multiple items, including physiological, morphological, and colloidal gold items. The supplementary provided with the instrument is designed for limited quantitation, which avoids unclear images due to excessive sampling volume and avoids the inability to chromatography during dripping specimens of colloidal gold items, improving accuracy. The bottom of the sample cup is equipped with a large area of longitudinal filter, which is conducive to easy filtration and precipitation of the formed elements; The bottom is also equipped with multi-level inclination and egg-collecting grooves, and the cup body is designed with an inverted triangle, which is conducive to the collection of the formed divided collection chamber and improves the recovery and detection rate. The KU-F40 AI deep learning algorithm automatically adds diluent according to the number of fecal specimens. Regarding the photo shooting technology, first uses a low-magnification lens to shoot the eggs, then the instrument locates the eggs, next the high-magnification lens automatically tracks and shoots, using multi-layer scanning imaging technology, the fecal specimens are automatically screened, and the positive specimens are transferred to manual assisted judgment and review.

In summary, the KU-F40 method has AI recognition characteristics, it is an ideal replacement for direct smear microscopy for routine screening because of its fast detection, simple operation, and high accuracy. Therefore, it has promotion and application value in the diagnosis of intestinal parasitic diseases.

## Acknowledgments

The authors thank the Zhuhai Keyu Bioengineering Co., Ltd., China, for providing *KU-F40 Fully Automatic Feces Analyzer* to enable parasite research.

## Author contributions

**Supervision:** Ximei Huang.

**Validation:** Lin Liao, Jinguang Tang.

**Writing – original draft:** Zila Wang.

**Writing – review & editing:** Faquan Lin.
